# Temperature Influences the Composition and Cytotoxicity of Extracellular Vesicles in Staphylococcus aureus

**DOI:** 10.1128/mSphere.00676-21

**Published:** 2021-10-06

**Authors:** Paul Briaud, Andrew Frey, Emily C. Marino, Raeven A. Bastock, Riley E. Zielinski, Richard E. Wiemels, Rebecca A. Keogh, Erin R. Murphy, Lindsey N. Shaw, Ronan K. Carroll

**Affiliations:** a Department of Biological Sciences, Ohio University, Athens, Ohio, USA; b Department of Cell Biology, Microbiology, and Molecular Biology, University of South Florida, Tampa, Florida, USA; c Heritage College of Osteopathic Medicine, Department of Biomedical Sciences, Ohio University, Athens, Ohio, USA; d Infectious and Tropical Disease Institute, Ohio University, Athens, Ohio, USA; University of Kentucky

**Keywords:** *Staphylococcus aureus*, temperature, extracellular vesicle, membrane vesicle, proteomics, transcriptomics

## Abstract

Staphylococcus aureus is a pathogenic bacterium but also a commensal of skin and anterior nares in humans. As S. aureus transits from skins/nares to inside the human body, it experiences changes in temperature. The production and content of S. aureus extracellular vesicles (EVs) have been increasingly studied over the past few years, and EVs are increasingly being recognized as important to the infectious process. Nonetheless, the impact of temperature variation on S. aureus EVs has not been studied in detail, as most reports that investigate EV cargoes and host cell interactions are performed using vesicles produced at 37°C. Here, we report that EVs in S. aureus differ in size and protein/RNA cargo depending on the growth temperature used. We demonstrate that the temperature-dependent regulation of vesicle production in S. aureus is mediated by the alpha phenol-soluble modulin peptides (αPSMs). Through proteomic analysis, we observed increased packaging of virulence factors at 40°C, whereas the EV proteome has greater diversity at 34°C. Similar to the protein content, we perform transcriptomic analysis and demonstrate that the RNA cargo also is impacted by temperature. Finally, we demonstrate greater αPSM- and alpha-toxin-mediated erythrocyte lysis with 40°C EVs, but 34°C EVs are more cytotoxic toward THP-1 cells. Together, our study demonstrates that small temperature variations have great impact on EV biogenesis and shape the interaction with host cells.

**IMPORTANCE** Extracellular vesicles (EVs) are lipid bilayer spheres that contain proteins, nucleic acids, and lipids secreted by bacteria. They are involved in Staphylococcus aureus infections, as they package virulence factors and deliver their contents inside host cells. The impact of temperature variations experienced by S. aureus during the infectious process on EVs is unknown. Here, we demonstrate the importance of temperature in vesicle production and packaging. High temperatures promote packaging of virulence factors and increase the protein and lipid concentration but reduce the overall RNA abundance and protein diversity in EVs. The importance of temperature changes is highlighted by the fact that EVs produced at low temperature are more toxic toward macrophages, whereas EVs produced at high temperature display more hemolysis toward erythrocytes. Our research brings new insights into temperature-dependent vesiculation and interaction with the host during S. aureus transition from colonization to virulence.

## INTRODUCTION

Extracellular vesicles (EVs) are membrane-derived spherical lipid bilayers theoretically secreted by all bacteria. During EV biogenesis, proteins and nucleic acids are packaged and released into the extracellular environment (1–4). Outer membrane vesicles (OMVs) secreted by Gram-negative bacteria were first described almost 60 years ago and have been implicated in biofilm formation, antibiotic resistance, virulence, and nucleic acid transfer (2). EVs from Gram-positive bacteria have been observed more recently and have features/functions similar to those of OMVs, with roles described in antibiotic resistance, virulence, functioning as decoys, and possessing immunomodulatory effects (1, 5–7).

Staphylococcus aureus is a skin and nose commensal bacterium but also a pathogen responsible for infections ranging from superficial skin disorders to life-threatening diseases. Although asymptomatic in the anterior nares, S. aureus increases the risk of invasive illnesses, and nasal strains are usually responsible for subsequent infections (8). Factors involved in the transition of S. aureus from commensal to pathogen are not fully understood, but temperature change (from lower temperatures to higher temperatures) is known to impact virulence gene expression in numerous bacterial species (9, 10). In S. aureus, we previously described an RNA element with temperature-responsive secondary structure (RNA-based thermosensor) involved in the temperature-dependent regulation of the *cidA* gene (11). This discovery led us to hypothesize that temperature change could play a role in S. aureus gene regulation. More recently, we showed that the temperature shift associated with the transition from commensal to pathogen (i.e., from 34°C to 37°C) is accompanied by widespread changes in transcriptome and proteome, demonstrating that temperature plays a much larger role than previously appreciated in regulating gene expression in S. aureus (11, 12).

EVs from S. aureus were the first vesicles described in a Gram-positive bacterium (13), and numerous studies have since characterized the protein content (or cargo) and interaction of S. aureus EVs with eukaryotic host cells during infections (1, 14–16). For instance, S. aureus vesicles are important in the development of atopic dermatitis (AD), a chronic inflammatory skin disease (17, 18). EVs containing the pore-forming toxin α-hemolysin induced increased necrosis and AD-like skin inflammation in mice compared to mice exposed to soluble α-hemolysin (17). Moreover, the complete cascade through which S. aureus EVs activate the inflammasome in macrophages was recently described and demonstrated how EVs function as an efficient virulence factor delivery system (14). Finally, the EV core proteome has been deduced by comparing EVs from different S. aureus isolates (both human and animal), characterizing in more detail the protein content and packaging in S. aureus (19).

Despite the recent advances in our understanding of S. aureus EVs, a number of important questions remain unanswered. The vast majority of studies on S. aureus EVs have been carried out using bacteria grown at 37°C, which corresponds to the internal temperature of the human body. Studies have also primarily concentrated on identification and characterization of EV protein cargo only. Very little is known about the impact of temperature on S. aureus EV production and content, limiting our understanding of the role of EVs during colonization (34°C) and the transition to pathogenic interaction (34°C to 37°C). Moreover, only one study to date has characterized the role of EV-associated nucleic acids during the infectious process, although the precise nucleic acid cargo of S. aureus EVs still remains unknown (20).

In this study, we demonstrate that EV production in S. aureus is temperature dependent, with temperature impacting both EV size and cargo. We demonstrate that the phenol-soluble modulin peptides (αPSMs) play an important role in the temperature-dependent release of EVs, which correlated with recently published data demonstrating increased αPSM production at higher temperatures. We go on to show that the protein and RNA cargo of EVs varies depending on growth temperature, with more protein and RNA packaged at lower temperature. Our data show that there is differential packaging of S. aureus virulence factors in EVs in a temperature-dependent manner, giving rise to EVs with differing phenotypic properties. EVs produced at higher temperature possess greater hemolytic activity, but EVs produced at low temperature display a greater cytotoxicity toward human monocytic cells.

## RESULTS

### Temperature influences αPSM-dependent EV production.

**FIG 1:**
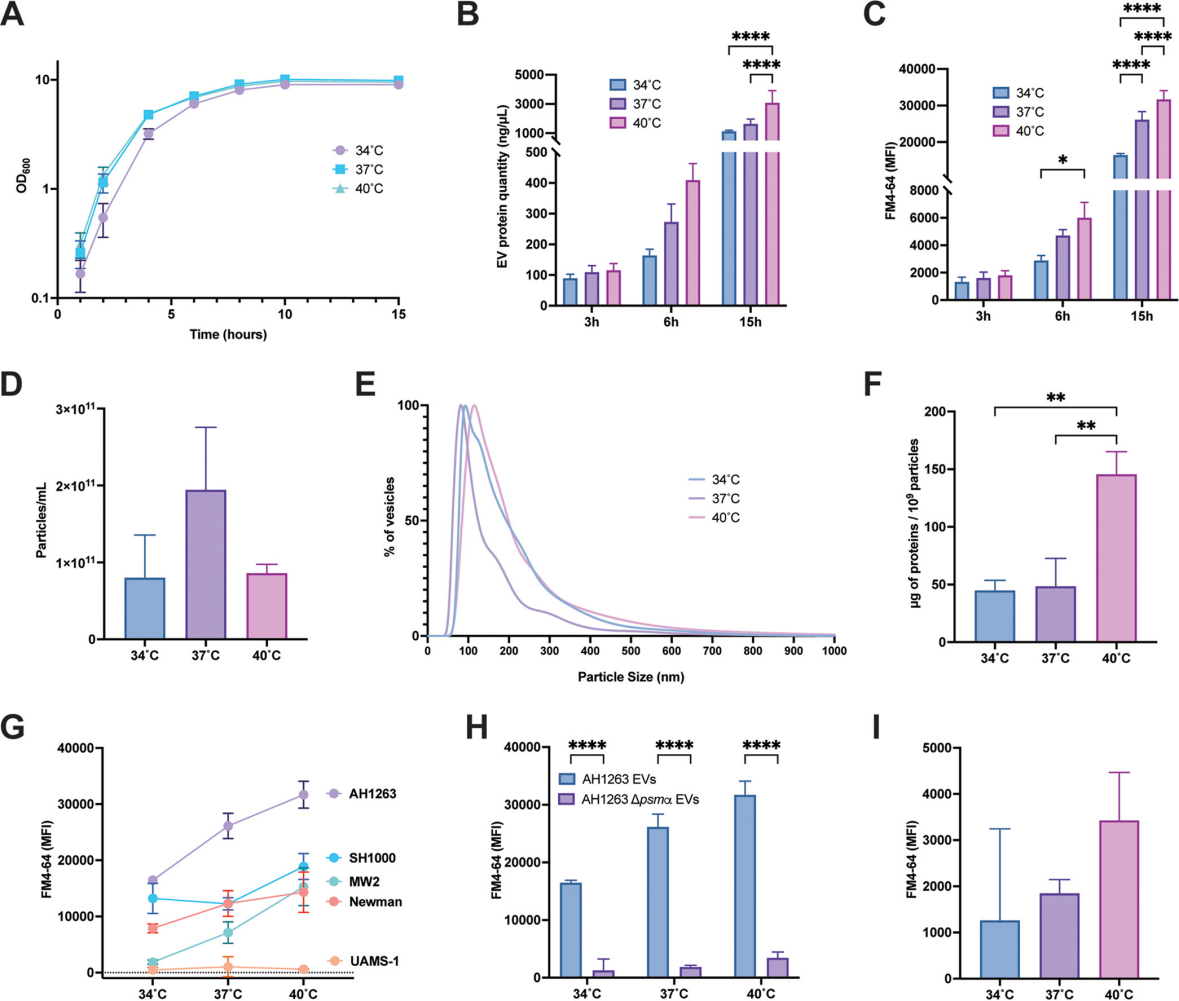
Temperature-dependent EV production in S. aureus. (A) Growth curves of wild-type AH1263 over 15 h in TSB at 34°C, 37°C, or 40°C. (B and C) Vesicles produced from AH1263 at 3 h, 6 h, and 15 h were analyzed for both protein (B) and lipid concentration (C). (D to F) Particle concentration (D) and size distribution (E) of S. aureus EV preparations were analyzed by NTA, and the relative concentration of proteins were estimated (F). (G) Vesicle production at 34°C, 37°C, and 40°C in different wild-type strains of S. aureus was analyzed by FM4-64. (H) EVs from the Δ*psmα* mutant were analyzed and compared to AH1263 EVs. The level of EV lipid from the Δ*psmα* mutant is indicated in more detail in panel I. Data represent means ± standard deviations (SD) from 3 independent experiments. *, *P* < 0.05; **, *P* < 0.01; ***, *P* < 0.001; ****, *P* < 0.0001, as calculated by analysis of variance (ANOVA) according to Materials and Methods.

S. aureus encounters temperature changes during the infectious process as it transitions from the nares and skin to deeper human tissues. Previous work in our lab examined the influence of temperature on S. aureus gene expression and revealed that modest temperature changes can have a dramatic impact on RNA and protein production (12). This study also demonstrated that αPSM production was temperature dependent, with increased αPSM production at higher temperatures (12). Since αPSMs are directly involved in EV release in S. aureus (21), we hypothesized that increased temperature would also positively impact EV release in an αPSM-dependent manner. To investigate this, S. aureus was grown at 34°C (skin/nasal colonization temperature), 37°C (human physiological temperature), and 40°C (pyrexia temperature) for 3 h, 6 h, or 15 h in tryptic soy broth (TSB). As previously observed by our group (12), other than an elongated lag phase, S. aureus grown at 34°C has no growth defect compared to cultures at 37°C and 40°C (Fig. 1A). EVs were isolated from S. aureus growing at 34°C, 37°C, and 40°C using a recently described precipitation method capable of obtaining high EV yields from lower volumes of culture compared to standard ultracentrifugation protocols (6, 21). EVs from each temperature were quantified by total protein concentration (Fig. 1B) and the amount of lipid (Fig. 1C). Proteins and lipids are major components of EVs, and their concentration has previously been shown to correlate to the level of EVs produced (6, 21). Results demonstrate that the amount of protein and lipid increases as the growth temperature rises, with the lowest protein and lipid concentration at 34°C and highest protein/lipid concentrations at 40°C. Temperature-dependent increases in protein/lipid concentration were observed in EV preparations from cultures grown for 6 h and 15 h but not for cultures at 3 h. Overall, an increase in EV production was also observed over time, with the highest levels of EVs isolated from cultures grown for 15 h. These results suggest (i) that EV production is growth phase dependent and/or that EVs accumulate in the media over time, and (ii) that S. aureus EV production is temperature dependent in a manner identical to αPSM production (12). Since the impact of temperature on vesicle production was greatest in 15-h cultures, all subsequent experiments were performed with EVs isolated at this time point.

Higher protein and lipid content at high temperatures could also be a consequence of larger vesicles being produced at these temperatures. To investigate this hypothesis, we performed a nanoparticle tracking analysis (NTA) to monitor particle concentration (Fig. 1D) and vesicle size at the different temperatures (Fig. 1E). Results show an increase in the number of vesicles produced at 37°C compared to 34°C (Fig. 1D). Although the difference observed is not statistically significant, this result could explain the increase in protein and lipid concentration determined by bicinchoninic acid (BCA) and FM4-64 between 34°C and 37°C (Fig. 1B and C). In contrast, there was no increase in vesicle concentration at 40°C compared to 37°C; in fact, a slight decrease in vesicle concentration was observed (Fig. 1D). This result indicates that the increase in protein/lipid concentration monitored in 40°C EV preparations is not due to an increase in the number of vesicles produced. Interestingly, size analysis showed that 37°C EVs are smaller in size than vesicles produced at 34°C or 40°C (Fig. 1E). EVs produced at 34°C and 40°C had similar size distributions, and each showed an increase in the number of larger vesicles compared to EVs at 37°C (Fig. 1E). When EV protein concentration was normalized by the number of EVs produced, results suggest that EVs produced at 40°C contain higher protein concentrations than vesicles produced at 34°C or 37°C (Fig. 1F). Collectively these results indicate that the difference in EV protein content at 34°C and 37°C can be explained by an increase in the number of vesicles produced; however, the increased protein content of EVs produced at 40°C is a result of more proteins being loaded.

To elucidate whether the impact of temperature on EV production was strain specific, we isolated EVs from 4 additional S. aureus strains: UAMS-1 (USA200) (22), Newman (23), MW2 (USA400) (24), and SH1000 (25). The temperature-dependent effect was observed in all strains tested, with the exception of UAMS-1, which is known to be a low αPSM producer (Fig. 1G) (26). This result indicates that the temperature-dependent effect is a well-conserved mechanism among αPSM-producing strains of S. aureus.

To further investigate the role of αPSMs in the temperature-dependent production of EVs in S. aureus, we isolated EVs from a *psmα* mutant at different temperatures and compared EV production to that of the wild type. As expected, EV production (measured by both protein and lipid concentration) is greatly reduced in the *psmα* mutant compared to the wild-type strain at all temperatures (Fig. 1H; see also Fig. S1A in the supplemental material). Interestingly, although EV production was much lower in the *psmα* mutant, a slight increase in EV lipid concentration was still observed at higher temperatures (Fig. 1I), corroborated by BCA assay for proteins (Fig. S1B). These results suggest that αPSMs are the major driver of temperature-dependent EV production but that other temperature-dependent factors may also play a minor role.

10.1128/mSphere.00676-21.1 FIG S1 Temperature-dependent EV production in the *psmα* mutant. Vesicles were isolated at 15 h from 34°C, 37°C, or 40°C and vesicles stained with the lipid dye FM4-64. Download FIG S1, PDF file, 0.4 MB. Copyright © 2021 Briaud et al. 2021 Briaud et al. https://creativecommons.org/licenses/by/4.0/ This content is distributed under the terms of the Creative Commons Attribution 4.0 International license.

### EV protein cargo is temperature dependent.

**FIG 2:**
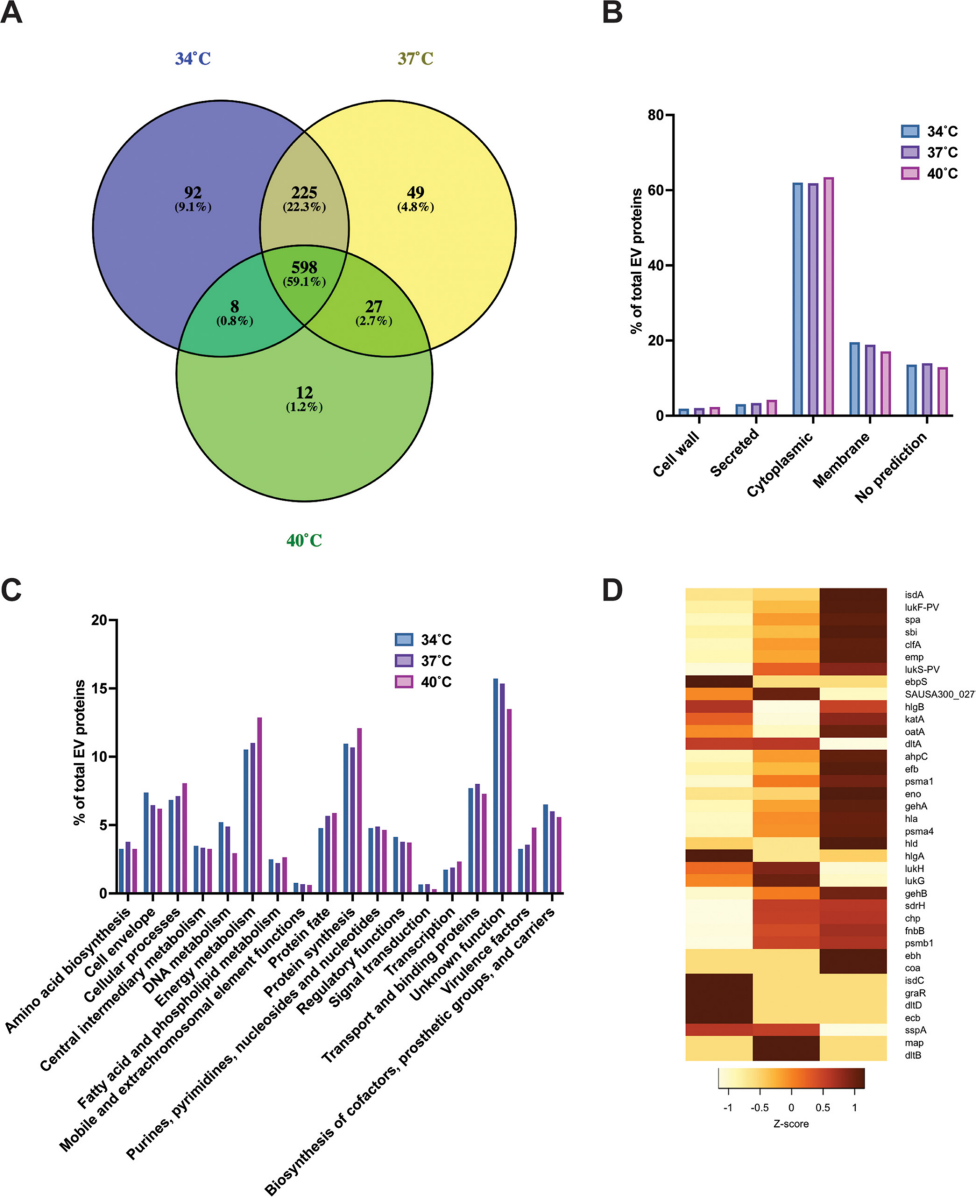
Temperature impacts S. aureus EV protein content. A mass spectrometry analysis was conducted on AH1263 EVs produced at 34°C, 37°C, and 40°C at 15 h. (A) Venn-diagram of EV proteins at each temperature. (B) EV protein distribution sorted by their predicted localization by PsortB. (C) Analysis and distribution of EV proteins by functional categories. (D) Heatmap depicting the Z-score of select virulence factors detected in EVs. A dark red color indicates a high abundance, whereas a light color depicts low abundance in EVs.

To gain better insight into the impact of temperature on EV biogenesis, mass spectrometry proteomic analysis was performed in triplicate to characterize the protein content of EVs produced at 34°C, 37°C, and 40°C (Data Set S1). A total of 598 unique proteins were identified in common between the three temperatures tested (Fig. 2A). A greater degree of similarity was observed in EV protein cargos at 34°C and 37°C (an additional 225 unique proteins in common) than between EV cargos at 34°C and 40°C (8 proteins in common) or 37°C and 40°C (27 unique proteins in common). A total of 923 unique proteins were detected at 34°C, 899 unique proteins at 37°C, and 645 unique proteins at 40°C, signifying that the protein diversity in vesicles is reduced when temperature increases. A similar pattern was observed for temperature-specific proteins, with more unique proteins observed at lower temperatures (92 proteins at 34°C versus 12 proteins at 40°C). Interestingly, 17% (*n* = 16) of unique proteins found only at 34°C belong to the functional category “cell envelope” (Data Set S1). In particular, these included 5 putative lipoproteins (Csa1A, SAUSA300_2355, SAUSA300_0102, SAUSA300_2430, and Ipl8) and 7 proteins involved in cell wall biosynthesis (TagB, TagX, MurG, MurC, MurE, TarL, and TarL′). At 37°C, 17% of unique proteins (*n* = 9) are associated with the functional category “transport and binding proteins.” Among the 12 proteins only detected at 40°C, 3 can be associated with S. aureus virulence (Ebh, Coa, and EsxA). The lipoprotein content in EVs follows the overall trend of proteins with more lipoproteins (*n* = 26) observed at 34°C than at 37°C (*n* = 22) or 40°C (*n* = 20) (Data Set S2). This suggests that temperature can impact the lipoprotein cargo of EVs. Of interest, the comparison of our analysis (15 h, 37°C) to previously published data (6 h, 37°C) (21) shows that most proteins found at 6 h (81%) are observed at 15 h (Data Set S3). This indicates that growth phase plays an important role in EV protein composition.

10.1128/mSphere.00676-21.3 DATA SET S1 Mass spectrometry proteomics data of EVs at 34°C, 37°C, and 40°C. Download Data Set S1, XLSX file, 0.4 MB. Copyright © 2021 Briaud et al. 2021 Briaud et al. https://creativecommons.org/licenses/by/4.0/ This content is distributed under the terms of the Creative Commons Attribution 4.0 International license.

10.1128/mSphere.00676-21.4 DATA SET S2 Lipoproteins detected in EVs at 34°C, 37°C, and 40°C. Download Data Set S2, XLSX file, 0.01 MB. Copyright © 2021 Briaud et al. 2021 Briaud et al. https://creativecommons.org/licenses/by/4.0/ This content is distributed under the terms of the Creative Commons Attribution 4.0 International license.

10.1128/mSphere.00676-21.5 DATA SET S3 Cross-reference analysis of proteins found in EVs in this study and by Schlatterer et al. (21). Download Data Set S3, XLSX file, 0.1 MB. Copyright © 2021 Briaud et al. 2021 Briaud et al. https://creativecommons.org/licenses/by/4.0/ This content is distributed under the terms of the Creative Commons Attribution 4.0 International license.

The predicted localization of EV proteins is similar between the different temperatures, with a high abundance of cytoplasmic proteins (about 60% of total proteins) followed by cytoplasmic membrane-associated proteins (about 15 to 20%) (Fig. 2B). Analysis of proteins by functional category (based on COGs [27]) showed a similar protein distribution across the different protein categories (Fig. 2C). However, an increase in proteins belonging to the categories energy metabolism, protein synthesis, and virulence factors can be seen at 40°C compared to other temperatures. Given the importance of virulence in S. aureus EV pathogenesis (13, 14, 19), we wanted to further explore vesicle-associated virulence factor composition at different temperatures. Among the 32 virulence factors identified in our data set, 24 are more abundant in EVs produced at 40°C (Fig. 2D). Well-characterized toxins involved in S. aureus virulence that were more abundant at 40°C are Hla (fold change at 40°C versus 34°C [FC 40vs34] = 1.91, Z-score = 1.02), αPSM (FC 40vs34 = 2.95, Z-score = 0.96), βPSM (not detected at FC 40vs37 = 6.95, Z-score = 0.67), and Hld (FC 40vs34 = 3.23, Z-score = 1.15). These results suggest that while the diversity of EV proteins is reduced at higher temperatures, more virulence factors are packaged at 40°C.

### Temperature impacts EV RNA cargo.

**FIG 3:**
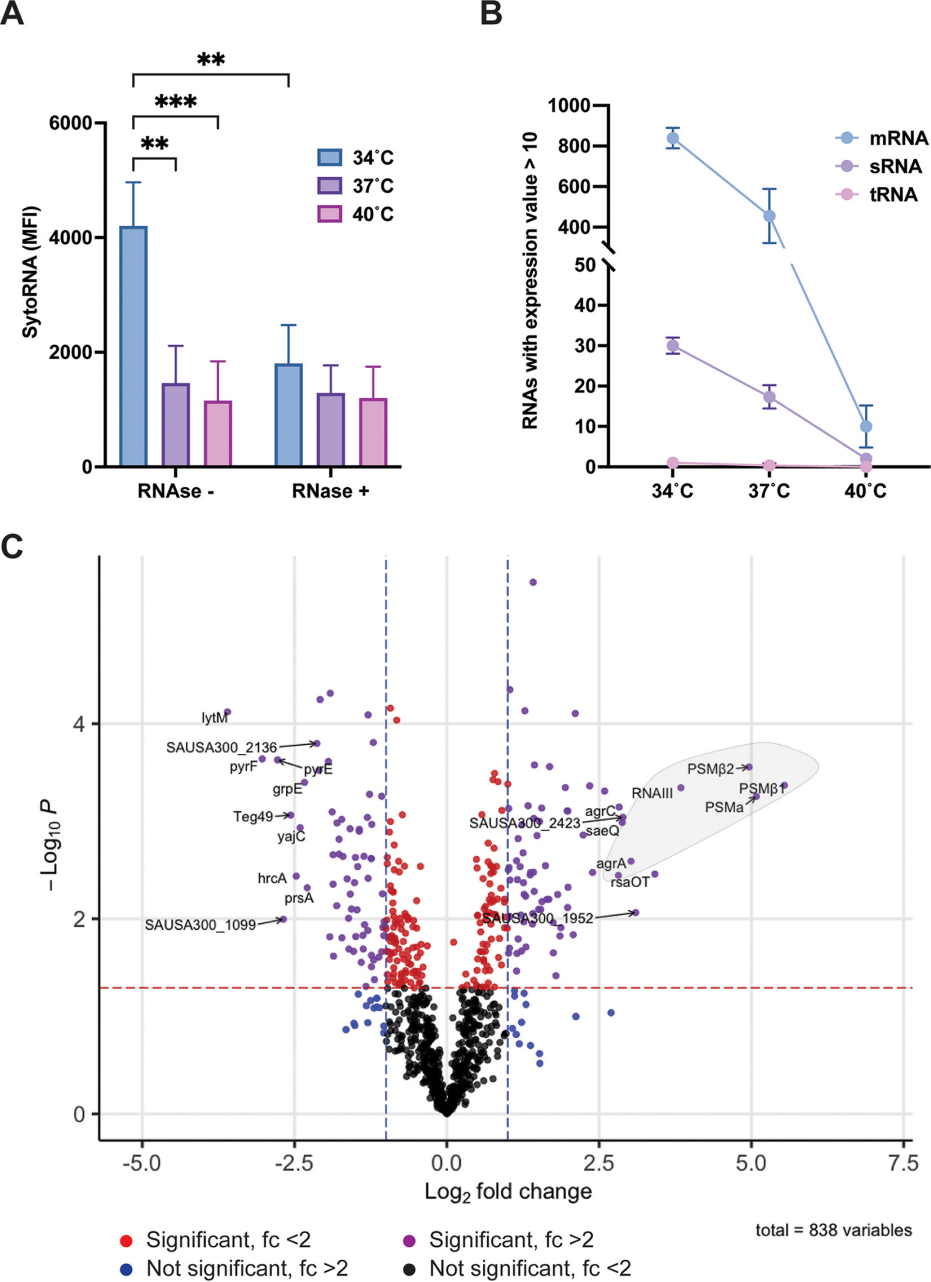
Temperature influences EV RNA composition. (A) The Syto RNAselect dye was used to quantify the amount of total RNA (surface and lumen associated; RNase^−^) or exclusively lumen-associated RNA (RNase^+^) in AH1263 EVs produced at 15 h at 34, 37, or 40°C. Data represent means ± SD from 3 independent experiments. **, *P* < 0.01; ***, *P* < 0.001, as calculated by ANOVA according to Materials and Methods. MFI, mean fluorescence intensity. (B) RNAs were sorted according to their classes in the cell (mRNA, sRNA, and tRNA). Only transcripts with mean expression values of >10 and >80% of unique mapped reads were used. (C) Volcano plot of differential expression analysis between 34°C and 37°C EV RNAs. Purple dots depict significant changes with log_2_ fold change of >1 and –log_10_*P* value of >1.3 (*P* < 0.05) cutoffs. Red and blue dots show transcripts reaching either significant *P* value (red dotted line) or log_2_ fold change (blue dotted lines) thresholds. Black dots indicate transcripts not differentially present. The first 10 differentially present transcripts under each condition are indicated. The bright gray circle points out the transcripts regulated by the Agr regulon.

Recent studies have demonstrated that in addition to proteins, nucleic acids, and, notably, RNAs, can be associated with S. aureus vesicles (20, 28). Furthermore, as reported by Rodriguez and Kuehn, EV-associated RNA can be located inside the lumen of the EV and/or associated with the external surface of the vesicle (20). To investigate the role of temperature in EV RNA composition, vesicles were isolated and incubated with/without RNase for 30 min prior to staining with the RNA-specific Syto RNAselect dye. The amount of RNA in the lumen, protected from RNase treatment, stayed unchanged across the three temperatures tested (Fig. 3A). However, RNase-sensitive, surface-associated RNA seemed to be more abundant at 34°C than at 37°C or 40°C. Furthermore, no difference in the SytoRNA fluorescent signal was observed between RNase^−^ and RNase^+^ EVs at 37°C and 40°C, suggesting that the majority of the EV RNA content is lumen associated at higher temperatures (Fig. 3A).

To better characterize the EV RNA content, transcriptome sequencing (RNA-seq) was performed in triplicate on RNA isolated from EVs produced at 34°C, 37°C, and 40°C (Data Set S4). We elected not to perform rRNA removal on RNA samples prior to RNA-seq, because at the time the experiment was performed the nature of the EV RNA (i.e., the relative amounts of rRNA, tRNA, sRNA, and mRNA) was unknown. A total of 3.24 × 10^6^ reads were sequenced from 34°C EVs and a total of 7.23 × 10^6^ reads from 37°C EVs (Table 1). Interestingly, only a small number of reads (1.17 × 10^3^) were sequenced from 40°C EVs (Data Set S4). Approximately 70% of reads from each sample could be mapped onto the USA300 reference genome. A large majority of mapped reads (94%, 97%, and 97% of the data sets at 34°C, 37°C, and 40°C) corresponded to rRNA transcripts (Table 1). This shows that rRNAs are highly abundant in S. aureus EVs. Moreover, the low number of reads sequenced at 40°C led us to hypothesize that high temperatures reduce the RNA cargo in EVs or lead to more rapid RNA degradation. To corroborate this hypothesis, we further examined the RNA composition (mRNA, sRNA, and tRNA) in EVs at the three temperatures. For this analysis, each RNA-seq data set was mapped to the USA300 genome and reads per kilobase per million (RPKM) expression values generated for each gene. To determine whether or not a transcript was present in EVs, we applied a threshold RPKM value of 10 (while this threshold is arbitrary, it was employed to eliminate genes that may have been represented in the data set by a very small number of reads). We also eliminated any genes where <80% of the reads mapped were not unique (nonunique reads typically correspond to repetitive sequences and/or duplicated genes and cannot definitively be mapped to their originating location on the genome). Once applied, these restrictions eliminated rRNA genes and allowed us to identify/enumerate the number of mRNA, sRNA, and tRNA genes represented in each sample. As depicted in Fig. 3B, mRNA represents the majority of EV RNA diversity, followed by sRNAs, while only a very small number of tRNAs can be detected. Interestingly, a clear trend is observed with a reduction in the overall number of mRNA and sRNA transcripts detected as temperature rises. These results strongly indicate that high temperatures decrease the RNA content (in terms of diversity) in S. aureus EVs.

**TABLE 1:** Summary of results of RNA-seq conducted on AH1263 EVs produced at three different temperatures

Read type	Value at^*a*^:		
	34°C	37°C	40°C
Total	3.24E + 06 ± 1.12E + 06 (100)	7.32E + 06 ± 2.78E + 06 (100)	1.17E + 03 ± 1.94E + 02 (100)
Mapped	2.39E + 06 ± 8.18E + 05 (73.7)	5.50E + 06 ± 2.05E + 06 (75)	8.61E + 02 ± 1.49E + 02 (73.7)
rRNA^*b*^	2.24E + 06 ± 7.60E + 05 (93.8)	5.34E + 06 ± 2.00E + 06 (97.2)	8.37E + 02 ± 1.54E + 02 (97.2)

a Results are shown as the means ± SD from triplicates. Percentages are indicated in parentheses.

b Percentage calculated from reads mapped.

10.1128/mSphere.00676-21.6 DATA SET S4 RNA-seq data of EV RNA at 34°C, 37°C, and 40°C. Download Data Set S4, XLSX file, 0.8 MB. Copyright © 2021 Briaud et al. 2021 Briaud et al. https://creativecommons.org/licenses/by/4.0/ This content is distributed under the terms of the Creative Commons Attribution 4.0 International license.

To further explore the variation in EV RNA content, a differential expression analysis (DEA) was performed to quantify changes in transcript levels between 34°C and 37°C EVs. The 40°C RNA-seq data were excluded from this analysis because of the low number of reads sequenced at this temperature and the low number of mRNA/sRNA transcripts represented (Fig. 3B). For the DEA, genes where <80% of the reads mapped were not unique were eliminated (as outlined above) and expression values at 34°C compared to those at 37°C. Raw expression values (in RPKM) were quantile normalized, and any gene(s) in which the normalized expression value at both temperatures was <10 was eliminated (as outlined above, this eliminated very weakly expressed/represented genes). The resulting data consisted of 838 transcripts that were identified in EVs at 34°C and 37°C and where expression under at least one condition was >10. We defined significant changes when the fold change between 37°C RNA EV versus 34°C RNA EV was >2 or <−2 (log_2_ fold change of >1 or <−1) and the *P* value was <0.05 (–log_10_*P* > 1.3). Seventy-three transcripts were more abundant in EVs at 37°C than 34°C, and 67 were more abundant at 34°C than 37°C (Fig. 3C). The 10 most abundant transcripts at 34°C are *lytM*, SAUSA300_2136, *pyrF*, *pyrE*, *grpE*, *yajC*, *hrcA*, *prsA*, SAUSA300_1099, and the sRNA *teg49*. Interestingly, at 37°C, 6 out of the 10 most abundant transcripts belong to the Agr regulon (*psmβ1*, *psmβ2*, *psmα*, *RNAIII*, *agrA*, and *agrC*). The 4 other transcripts enriched at 37°C are *saeQ*, SAUSA300_2423, SAUSA300_1952, and *rsaOT* (Fig. 3C). Collectively, our results confirm that RNAs can be found in S. aureus EVs and show that temperature impacts the vesicle RNA cargo.

### EV cytotoxicity is temperature dependent.

**FIG 4:**
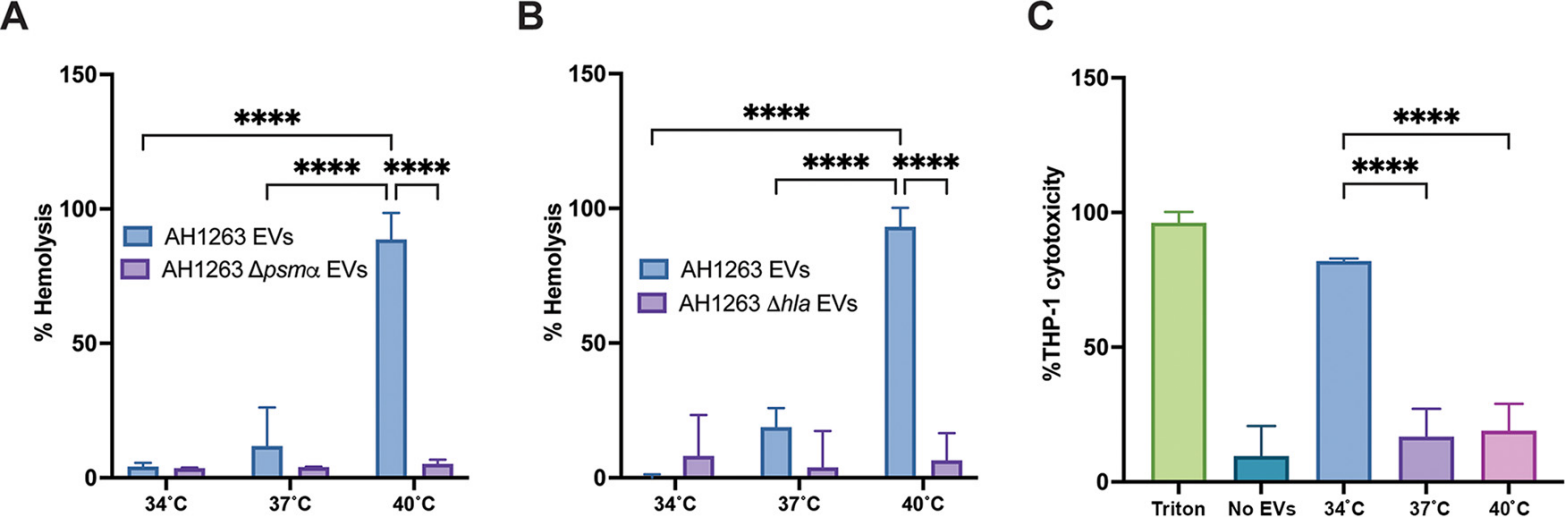
EV cytotoxicity is temperature dependent. Human (A) or rabbit (B) erythrocyte hemolysis was assessed by incubating 20 μg of EVs produced at each temperature for either 15 min (for human blood) or 2.5 min (for rabbit blood) at 37°C. The hemolytic activity was determined by reading the absorbance of the samples at OD_543._ (C) THP-1 cells were incubated with 5 μg of EVs for 4 h prior to cell viability assessment. Data represent means ± SD from 3 independent experiments. ****, *P* < 0.0001 as calculated by analysis of variance (ANOVA) according to Materials and Methods.

To date, the cytotoxicity of S. aureus vesicles has only been studied from cultures grown at 37°C. Our results, demonstrating increased packaging of virulence factors at higher temperatures, led us to hypothesize that EV cytotoxicity toward host cells also is temperature dependent. To test this hypothesis, the hemolytic activity of vesicles was monitored toward both human erythrocytes (αPSM-mediated hemolysis) and rabbit erythrocytes (Hla-mediated hemolysis). As shown in Fig. 4A, the highest hemolytic activity toward human red blood cells was observed in EVs produced at 40°C, consistent with our previous results showing increased αPSM production at higher temperatures (12) and the data presented here showing increased packaging of αPSMs at higher temperature (Fig. 2D). To confirm the involvement of αPSMs, the hemolysis assay was repeated with vesicles produced from the Δ*psmα* mutant at the three temperatures. The absence of *psmα* completely abrogated hemolysis from vesicles (Fig. 4A), confirming the increase in hemolysis observed in 40°C EVs was αPSM dependent. Similarly, we observed greater hemolysis toward rabbit erythrocytes using 40°C EVs (Fig. 4B), consistent with the increased abundance of Hla at this temperature (Fig. 2D). A strain carrying a transposon insertion in the *hla* gene, which is not impacted in vesicle production (Fig. S2), showed no hemolytic activity, confirming Hla as the toxin primarily responsible for rabbit erythrocyte hemolysis (Fig. 4B).

10.1128/mSphere.00676-21.2 FIG S2 Temperature-dependent EV production in the *hla* mutant. Vesicles were isolated at 15 h from 34°C, 37°C, or 40°C and vesicles quantified by protein concentration. Download FIG S2, PDF file, 0.3 MB. Copyright © 2021 Briaud et al. 2021 Briaud et al. https://creativecommons.org/licenses/by/4.0/ This content is distributed under the terms of the Creative Commons Attribution 4.0 International license.

**FIG 5:**
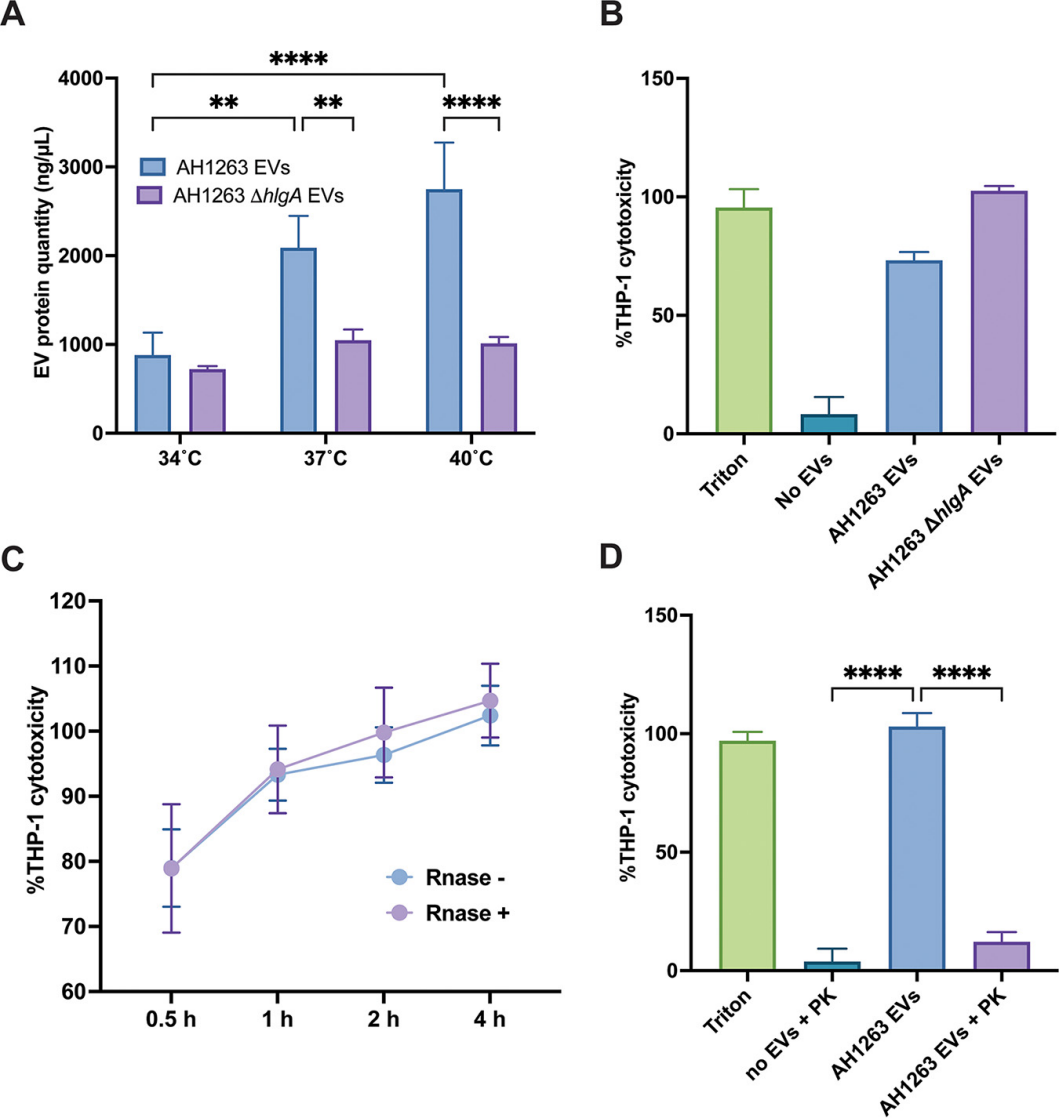
Surface-associated proteins are responsible for cytotoxicity of 34°C EVs. (A) Vesicles produced at 15 h from AH1263 WT and AH1263 carrying a transposon inserted into the *hlgA* gene (*hlgA*) were analyzed for protein content. (B to D) THP-1 cells were exposed to 5 μg of either *hlgA* mutant EVs (B) for 4 h, AH1263 EVs (with or without RNase) for 0.5 h, 1 h, 2 h, or 4 h (C), or proteinase K-treated EVs for 4 h (D) prior to cell viability assessment. Data represent means ± SD from 3 independent experiments. ****, *P* < 0.0001 as calculated by analysis of variance (ANOVA) according to Materials and Methods.

We next wanted to investigate the cytotoxicity of EVs using human monocyte/macrophage THP-1 cells. Cells were incubated with EVs produced at the three different temperatures for 4 h before monitoring the cell viability by 3-(4,5-dimethyl-2-thiazolyl)-2,5-diphenyl-2H-tetrazolium bromide (MTT) assay. Surprisingly, EVs generated at 34°C showed greater cytotoxicity toward THP-1 cells than EVs generated at 37°C or 40°C (Fig. 4C). This outcome is the opposite of our anticipated result, based upon our observation of greater virulence factor packaging at 40°C and the increased hemolysis activity reported in human blood at 40°C. To understand the increased cytotoxicity observed toward THP-1 cells at 34°C, we examined the mass spectrometry data for proteins that were more abundant at lower temperatures. Interestingly, results show the gamma hemolysin component A, HlgA, is more abundant in EVs at 34°C than 37°C or 40°C (Fig. 2D), which could explain the greater cytotoxicity observed at this temperature. HlgA is one subunit of a bicomponent pore-forming toxin and attaches to CCR2, CXCR2, and DARC host cell receptors. HlgA binding leads to lysis of host cells by recruiting the gamma hemolysin component B, HlgB (29). To test the hypothesis that increased EV cytotoxicity at 34°C is mediated by HlgA, we first produced EVs from an *hlgA* mutant strain and monitored vesicle production compared to that of the wild type. Interestingly, vesicle production in the *hlgA* mutant strain was impacted at 37°C and 40°C but not at 34°C, suggesting a role for HlgA in vesicle production at 37°C and 40°C (Fig. 5A). To test the cytotoxicity of *hlgA* mutant-derived EVs, we exposed THP-1 cells to EVs produced at 34°C from the wild-type and *hlgA* mutant strains and monitored the cytotoxicity at 4 h (Fig. 5B). Cytotoxicity of *hlgA* mutant EVs was similar to that of wild-type (AH1263) EVs, showing that the increased cytotoxicity of S. aureus EVs at 34°C is not HlgA mediated.

Another notable characteristic of S. aureus EVs at 34°C is the high abundance of EV surface-associated RNA. Having ruled out a role for HlgA in the increased cytotoxicity of EVs at 34°C, we hypothesized that the surface-associated RNA could be involved in the cytotoxicity of vesicles. To test this, we exposed THP-1 cells to RNase-treated 34°C EVs and monitored the cytotoxicity over 4 h (Fig. 5C). However, no difference was observed between RNase-treated (RNase^+^) and nontreated (RNase^−^) EVs, eliminating the surface-associated RNA hypothesis. Finally, we treated 34°C EVs with proteinase K for 30 min prior to THP-1 cell exposure to determine if EV surface-associated proteins are responsible for the increased cytotoxicity at 34°C. Interestingly, the depletion of EV surface proteins abrogated THP-1 cytotoxicity (Fig. 5D). This result indicates that the increased cytotoxicity observed in 34°C EVs is mediated by proteinase K-sensitive surface-exposed protein(s). Collectively, our results show that temperature impacts EVs cytotoxicity in a protein-dependent fashion.

## DISCUSSION

Extracellular vesicles from Gram-positive bacteria have long been overlooked; however, work over the last 10 years has begun to explore this new aspect of Gram-positive physiology. The involvement of vesicles in S. aureus pathogenesis has become clear, as numerous studies describe interactions between S. aureus and host cells mediated by EVs (1). The content of EVs, i.e., proteins, nucleic acids, and lipids, is intrinsically linked to metabolism of the bacterium, as vesicles are packaged and derive from the cytoplasm and the bacterial membrane. Almost all studies performed in S. aureus to date have examined EV production at 37°C, the physiological temperature of the interior of the human body. However, 37°C is not the only temperature experienced by S. aureus and, in fact, may not be the most common temperature experienced. S. aureus living as a commensal (on the human skin and anterior nares) likely experiences temperatures slightly lower than core body temperature (approximately 34°C); consequently, studies of EVs at 37°C disregard the impact of other relevant temperatures in vesiculogenesis. Our study reports the impact of temperature on EV cargo and production and clearly demonstrates the importance of temperature in host cell-EV interactions.

We observed that temperature altered vesicle production and morphology in S. aureus, with more vesicles produced at 37°C but larger-sized vesicles produced at 34°C and 40°C. This result can be explained in part by our recent finding that the αPSM peptides (known stimulators of EV biogenesis) are also produced in a temperature-dependent manner (12). In Gram-negative bacteria, temperature-dependent vesiculation is species specific. In Escherichia coli, vesiculation increases with temperature, but in *Serratia marcenscens*, lower temperatures result in increased vesicle production (3). Time of EV isolation is also an important factor, with a time-dependent increase in lipid and protein content in EVs. The impact of temperature on EVs is a shared mechanism among different strains of S. aureus. To our knowledge, only one other study has examined EV production in S. aureus at different temperatures. Wang et al. (30) recently demonstrated EV protein cargo increased at 30°C compared to that at 37°C or 40°C, while no difference was observed between 37°C and 40°C. These results are in contrast to the results observed in this study. The reason for the discrepancy in results between our data and that of Wang et al. is not immediately clear; however, one notable difference is the low temperatures used in both studies. Here, we use 34°C (similar to our previous analysis [12]), while Wang et al. used 30°C. We have previously demonstrated that S. aureus physiology can be dramatically altered by changes in temperature as small as 4°C; therefore, this difference in temperature may be responsible for the variation observed. Differences in strains used and time of vesicle isolation could also be responsible for the discrepancies.

Our results with the *psm*α mutant (Fig. 1) implicate αPSMs as the main driver of temperature-dependent vesicle production in S. aureus. This finding is consistent with our recent findings that S. aureus αPSM production is temperature dependent (12) and with the findings of Schalterrer et al., who demonstrated that αPSMs are involved in vesicle production in S. aureus by increasing membrane fluidity (21). Nonetheless, other factors appear to be involved, as illustrated by the slight increase of vesicle production in the *psm*α mutant. It is possible that a direct influence of temperature on membrane fluidity could be responsible for this effect. The impact of vesicle production at high temperatures could represent a disposal mechanism through which the cell jettisons misfolded proteins that accumulate at high temperatures, preventing their toxic accumulation in the cell. EVs at 40°C are more densely loaded with proteins than EVs at other temperatures, supporting of this potential disposal mechanism.

Our proteomic analysis identified increased numbers of proteins in S. aureus EVs compared to previous studies (13, 19, 21). Many of the proteins identified in our analysis were previously shown by others to be found in EVs, but we also identified many proteins for the first time in S. aureus EVs. Vesicle production and composition in S. aureus are known to be strain dependent (19); however, the primary reason underlying the increased number of proteins in our study is likely because we isolated EVs during the stationary phase. Previous studies have looked at EV proteins from early- to mid-exponential-phase cultures (13, 21, 31), and our comparison with a previous study (21) showed that most proteins isolated at 6 h are found in EVs at 15 h. It is likely that stationary-phase proteins packaged in EVs were detected in our analysis, explaining the large number of proteins detected. Interestingly, as temperatures rise, the number of individual proteins packaged in EVs decreases. This finding supports our recent observation that a large number of S. aureus proteins display decreased abundance as temperature increases (12). Increased temperature may have a negative impact on protein translation in S. aureus and/or decrease the half-life of proteins and, therefore, could explain the decreased number of proteins in the EV cargo. We also observed temperature-dependent packaging of virulence factors, including both subunits of the Panton Valentine leucotoxin (LukF/S-PV), αPSM, βPSM, Hld, and Hla, all of which were more abundant at 40°C (the opposite of the overall trend for EV proteins). The increased packaging of virulence factors at higher temperatures suggests vesicles produced at high temperatures can participate in S. aureus infection. Moreover, the increased abundance of virulence factors in EVs at 40°C (a temperature associated with fever) indicates a role for EVs in resisting the immune response to infection. On the other hand, at 34°C the decreased abundance of virulence factors in vesicles could facilitate the colonization lifestyle of the bacterium. Further studies are necessary to investigate the role of EVs in the transition between colonization and infection.

The presence of RNA in S. aureus vesicles was described for the first time in 2020 (20), and very recently, the first RNA-seq analyses were made from S. aureus EVs grown under stress condition at different time points (28, 32). Although the conditions under which we performed EV RNA-seq were different (i.e., the medium and strains used), we observed a similar RNA packaging pattern, with most of the RNAs detected encoding proteins. A total of 838 RNAs were detected under our conditions in EVs, more than previously described (28). Our study confirms the presence of RNAs encoding virulence factors in EVs and shows temperature-dependent packaging of transcripts, with more RNAs encoding virulence factors packaged at 37°C than 34°C (*hld*, α*psm*, *psm*β1, *psm*β2, *agrA*, *agrB*, *agrC*, *agrD*, and *sbi*). Thirty sRNA transcripts also were found at 34°C and 37°C, and 6 of them showed differential packaging according to temperature, with Teg49 being more abundant at 34°C and RNAIII, RsaC, RsaOT, SAUSA300s005, and SAUSA300s003 being more packaged at 37°C. Interestingly, RNAIII and RsaC were identified here and also described in the two previous studies and, therefore, may be important in vesicle-host interactions (28, 32). At 40°C, the RNA content in EVs was dramatically decreased, with only approximately one thousand reads sequenced. The SytoRNA staining revealed the presence of RNA at 40°C, which means that RNAs are still present at 40°C According to our RNA-seq data, the majority of RNA present at 40°C are rRNA (Data Set S4). It is still unclear if rRNAs are selectively packaged at 40°C or RNAs are more degraded in EVs at this temperature.

A similar temperature-responsive pattern can be observed from our proteomic and transcriptomic data, with decreased RNA and proteins in EVs as temperature increases. The mechanism through which temperature influences the EV composition remains unknown, but two main hypotheses can be made. First, EV cargo could directly reflect the impact of temperature on cell physiology and, therefore, the temperature passively impacts vesicle packaging. Alternatively, temperature could actively impact the packaging process of vesicles during their formation. Schlatterer et al. noted that the protein composition of vesicles from the *psmα* mutant was different from that of wild-type vesicles (21). Given the importance of the αPSM in temperature-dependent vesiculogenesis, it is tempting to speculate that temperature impacts EV packaging (proteins and/or RNAs) through αPSM production.

In addition to influencing EV cargo, our findings show that temperature influences the cytotoxicity of S. aureus EVs toward eukaryotic cells. At 40°C, EVs displayed the highest levels of hemolytic activity toward human and rabbit erythrocytes, and we confirmed the role of αPSMs and Hla in these phenotypes. Surprisingly, 34°C EVs displayed the highest levels of cytotoxicity toward THP-1 cells despite the high virulence factor content at high temperatures detected by mass spectrometry. With the exception of HlgA, which we demonstrate is not responsible, our proteomic data do not show any obvious candidates (e.g., toxins/virulence factors); however, we cannot rule out the possibility of a moonlighting protein or protein of unknown function being responsible. Once delivered inside cells, bacterial proteins may have roles other than their original function in the bacterium that may participate in host cell death. Another hypothesis is that temperature could impact the protein localization (lumen or surface) in S. aureus EVs and dictates the cytotoxicity toward THP-1 cells. Due to its increased abundance in EVs at lower temperatures, we also investigated whether surface-exposed RNA could be responsible for the increased cytotoxicity at 34°C; however, depleting these RNase-sensitive RNAs did not impact the cytotoxicity of 34°C EVs. Nonetheless, we cannot exclude that lumen RNase-resistant RNAs could play a role in EV cytotoxicity. Recent reports describe that bacterial RNAs are potent inducers of the immune response in eukaryotic cells through recognition by Toll-like receptors and that S. aureus EV RNAs are delivered inside host cells (20, 33–35). Direct base pairing between bacterial RNAs and eukaryotic RNAs may occur, as described in Gram-negative bacteria, and could hijack host cell responses and lead to the cytotoxicity observed (36). Furthermore, the high percentage of cytotoxicity observed (80% of cell mortality after 30 min of incubation) led us to hypothesize that 34°C vesicles may be internalized faster than vesicles produced at 37°C or 40°C. Previous studies have shown that S. aureus EVs can be internalized either via a dynamin-dependent endocytosis or by direct fusion with cholesterol-rich membrane microdomains (14, 31). Interestingly, 34°C EVs display more unique lipoproteins (Csa1A, SAUSA300_2355, SAUSA300_0102, SAUSA300_2430, and Ipl8), which could promote faster internalization and delivery of protein/RNA cargoes inside the host cells. Previous studies suggested that EV-associated virulence factors need to be delivered inside host cells to be potent (17, 31, 37). Thus, a more efficient internalization of EVs could lead to greater cytotoxicity. Work is currently ongoing in our lab to determine the factors/processes that lead to increased cytotoxicity of 34°C EVs.

The study presented describes, for the first time, the impact of physiologically relevant temperatures on S. aureus EV biogenesis and composition. It highlights the important role small temperature variations play in S. aureus physiology and interactions with host cells. High temperatures lead to increased packaging of virulence factors in vesicles and suggest they play important roles in the establishment of acute/systemic infection. Although further studies are required to fully understand temperature-dependent EV packaging and interaction with the host, our report shed lights on the importance of environmental factors in vesiculogenesis and the potential impact of vesicles in the transition from commensal to virulence.

## MATERIALS AND METHODS

### Strains and growth conditions.

S. aureus strains used in this study are listed in Table 2. Strains were grown at 37°C in tryptic soy broth (TSB) with shaking at 200 rpm overnight before being inoculated in prewarmed TSB at 34°C, 37°C, or 40°C. The *hlgA*::bursa (*hlgA* mutant) strain was constructed via phage transduction (38) from strain NE1399, which was obtained from the Network on Antimicrobial Resistance in Staphylococcus aureus (NARSA) transposon mutant library (39).

**TABLE 2:** Strains used in the study

S. aureus strain	Description	Reference or source
AH1263	USA300 LAC isolate cured of plasmid LAC-p03	46
SH1000	NCTC 8325 with *rsbu* repaired	25
MW2	USA400, septicemia isolate	24
Newman	Laboratory strain	23
UAMS-1	MSSA osteomyelitis isolate	22
RKC1079	AH1263 *hlgA*::ery, *hlgA* mutant	This study
RKC0753	AH1263 Δ*psmɑ*	47
NE1399	USA300 JE2 *hlgA*::*ery* NARSA transposon mutant	39

### Extracellular vesicle isolation.

Extracellular vesicles were isolated as previously described (21). Briefly, S. aureus cultures were centrifuged for 15 min at 3,000 × *g*. Supernatants were filter sterilized through 0.22-μm membranes, and 15 ml of filtered-sterilized supernatant was concentrated using 100-kDa concentrators (MAP100C37; Pall). ExoQuick-TC buffer (ExoTC20A-1; System Bioscience) was added (1/5 of total volume) to the retentates and incubated overnight on ice at 4°C. Mixtures were centrifuged at 1,500 × *g* for 30 min at 4°C, and vesicle pellets were resuspended in 500 μl of phosphate-buffered saline (PBS).

### Vesicular protein, lipid, RNA, and size characterization.

Vesicle protein concentration was determined from 25 μl of the 500 μl obtained from the previous step by using the Pierce BCA protein assay kit (23225; Thermo Scientific) according to the manufacturer’s instructions. For lipids, vesicles (25 μl) were stained in 5 μg/ml FM4-64 lipid dye (T3166; Invitrogen) and incubated for 20 min at 37°C. For RNA, the same amounts of EV proteins (5 μg) were treated with 1 U of DNase (AM2238; Invitrogen) and, if indicated, with 10 μg/ml of RNase A (19101; Qiagen) in 100 μl of PBS for 30 min at 37°C. Next, 1 mM Syto RNASelect (S32703; Invitrogen) was added, and samples were incubated for 10 min at room temperature. Fluorescence was read at 740 nm for lipids or 530 nm for RNA with a plate reader (Synergy HTX; Biotek). Size determination and particle counts were performed as outlined in reference 21, using dynamic light scattering analysis with an LM10 NanoSight (Malvern Panalytical) according to the manufacturer’s instructions.

### Cell-free erythrocyte hemolysis assay.

Hemolytic activity of EVs was performed as previously described, with minor modifications (40). EV suspensions (100 μl at 200 μg/ml) were diluted 1:2 in reaction buffer (40 mM CaCl_2_, 1.7% NaCl) and incubated at 37°C in a tube revolver with 25 μl of whole blood from humans or rabbits. After 2.5 min (rabbit) or 15 min (human), the samples were centrifuged at 5,500 × *g*, and 100 μl of supernatant was transferred to a 96-well plate. The hemolytic activity was determined by reading the absorbance of the samples at the optical density at 543 nm (OD_543_). The percentage of hemolysis was calculated by setting the highest OD_543_ as 100% and the OD_543_ from negative controls (no EVs) as 0% hemolysis.

### Cytotoxicity assay.

The human monocytic cell line (THP-1) was routinely maintained in Roswell Park Memorial Institute medium (RPMI 1640) supplemented with 10% fetal bovine serum (FBS) and a cocktail of 1% penicillin-streptomycin. THP-1 assays were based on the previously published invasion assay (41). Macrophages were seeded at a density of 2 × 10^5^ cells/well. When indicated, cells were exposed to 5 μg of EVs for 0.5, 1 h, 2 h, or 4 h. Macrophages were washed 3 times with PBS, and cell viability was assessed by using the Vybrant MTT cell proliferation assay kit (ThermoFisher) according to the manufacturer’s instructions. Cells treated with PBS only were used as a negative lysis control, while cells treated with Triton X-100 were used as a positive lysis control.

### Proteomic analysis.

A volume corresponding to 50 μg of EV protein was precipitated by using the ExoQuick-TC buffer. EV pellets were prepared using S-trap microcolumns (Protifi) according to the manufacturer’s instructions. Briefly, samples were resuspended in 5% (wt/vol) SDS, 10 mM dithiothreitol, 100 mM triethylammonium bicarbonate (TEAB) with protease inhibitor cocktail (ThermoFisher Scientific), reduced for 10 min at 95°C, and clarified by centrifugation at 17,000 × *g* for 10 min. Protein concentration was determined by Pierce 660-nm protein assay (ThermoFisher Scientific) and samples standardized to 50 μg, reduced with 30 mM iodoacetamide for 30 min in the dark at room temperature, quenched with 1.2% (vol/vol) phosphoric acid, combined with S-trap buffer (100 mM TEAB, pH 7.4, in 90% [vol/vol] methanol), and adsorbed to the S-trap columns and washed according to the manufacturer’s protocol. Digestion was performed using 1 μg trypsin in TEAB and incubated at 37°C for 18 h. Samples were eluted, desiccated, and resuspended in 1% acetonitrile, 0.1% formic acid. Trypsin-digested peptides (5 μl) were separated on a 50-cm Acclaim PepMap 100 C_18_ reverse-phase high-pressure liquid chromatography (HPLC) column (Thermo Fisher Scientific) using an Ultimate3000 UHPLC (Thermo Fisher Scientific) with 180-min gradient (2% to 32% acetonitrile with 0.1% formic acid). Peptides were analyzed on a hybrid Quadrupole-Orbitrap instrument (Q Exactive Plus; Thermo Fisher Scientific) using data-dependent acquisition in which the top 10 most abundant ions were selected for tandem mass spectrometry analysis. Raw files were searched against the S. aureus USA300 proteome (UniProt ID UP000001939) using MaxQuant (www.maxquant.org) and the integrated Andromeda search engine. Digestion was set as trypsin/P, variable modifications included oxidation (M) and acetylation (protein N-terminal), and carbamidomethylation (C) was fixed. Label-free quantification was used, with peptides matched between runs. Other settings were left as defaults. Missing values in the resulting file were replaced with ones. The Z-score was calculated for virulence factors detected in vesicles and used to display a heatmap with RStudio V1.4.1106 (42) and the gplots package (V3.1.1).

### EV RNA extraction.

EV RNA extraction was performed using the miRNeasy minikit (217004; Qiagen) to maximize sRNA recovery. EV pellets precipitated by ExoQuick-TC buffer were resuspended in 260 μl of buffer RLT containing 10 μl of β-mercaptoethanol and mixed thoroughly for 30 s. Next, 80 μl of buffer AL was added to each sample and transferred to genomic DNA column eliminators. One volume of isopropanol was added to the flowthrough, and samples were transferred to RNeasy Mini columns and centrifuged for 15 s at 8,000 × *g*. Columns were sequentially washed with buffer RWT, RPE, and 80% ethanol according to the manufacturer’s instructions. EV RNAs were eluted in 30 μl of nucleases-free water. Finally, samples were treated with Turbo DNase (AM2238; Invitrogen) for 30 min at 37°C to remove DNA contamination. RNA profiles and concentrations were assessed by using the RNA pico chips (5067-1513; Agilent Technologies) and Agilent 2100 Bioanalyzer.

### RNA-seq analysis.

RNA-seq was carried out by the Microbial Genome Sequencing Center (MiGS) (Pittsburgh, PA). Libraries were created using the QIAseq FX single-cell RNA library kit. Sequencing reactions were carried out on an Illumina NextSeq 550 with a 75cyc high-output flowcell. Data were exported and analyzed using CLC Genomics Workbench as described previously (43). To sort RNAs according to their classes in the cell (mRNA, sRNA, and tRNA), individual data sets were exported and cutoffs were applied (RPKM expression value of >10, and >80% of mapped reads are unique). Each feature identifier then was associated with its class of molecule by using the *Aureo*Wiki database (44). For the differential expression analysis, the 34°C and 37°C data sets were quantile normalized and cutoffs were applied (>80% of mapped reads are unique). Genes that had normalized expression values of <10 under both conditions (i.e., 34°C and 37°C) were excluded from further analysis, leaving only genes where the expression value was >10 under at least one condition. The resulting data were used to generate a volcano plot by using Rstudio V1.4.1106 (42) and the EnhancedVolcano package V1.8.0 with cutoffs set at a log_2_ fold change of >1 and −log_10_*P* value of >1.3 (*P* < 0.05).

### Statistical analyses.

Statistical analyses were performed with GraphPad Prism 9, with a *P* value of <0.05 considered significant. Analysis of variance (ANOVA; one way or two way depending on whether results were grouped) with Tukey’s multiple-comparison test was used.

### Ethics statement.

Human blood samples were obtained from healthy donors at Ohio University. All collections, handling, and usage of blood was approved by the Ohio University Institutional Review Board protocol number 17-X-79. Rabbit blood was purchased from Hemostat Laboratories.

### Data availability.

The mass spectrometry proteomics data have been deposited in the ProteomeXchange Consortium via the PRIDE (45) partner repository with the data set identifier PXD025236. RNA-seq data were deposited in the Gene Expression Omnibus (GEO) database under the accession number GSE171489.
