# Supplementary figures and images for: Temperature Influences the Composition and Cytotoxicity of Extracellular Vesicles in Staphylococcus aureus
Source: mSphere. 2021 Oct 6;6(5):e00676-21. doi: 10.1128/mSphere.00676-21 (PMC8510519; doi:10.1128/mSphere.00676-21)

**A**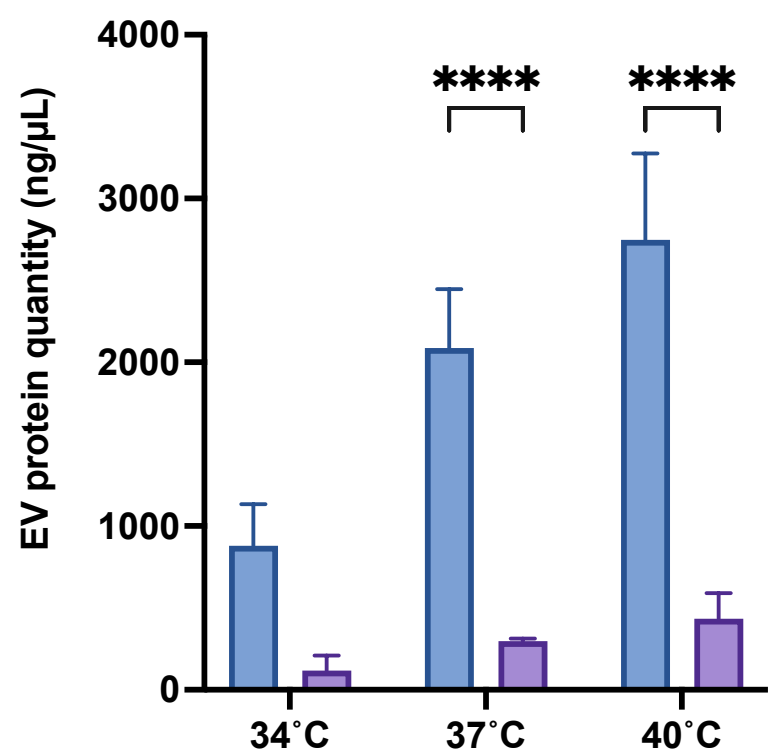**B**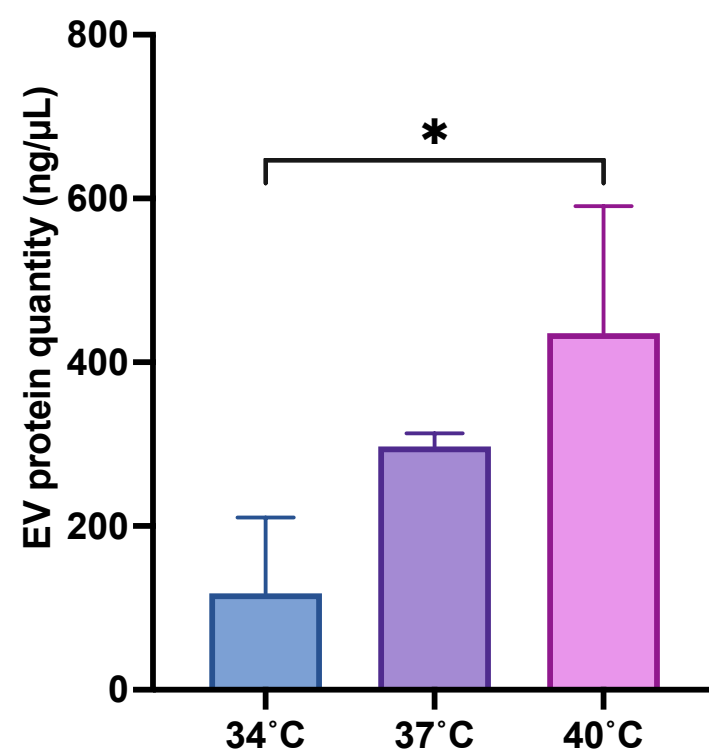

Supplement: FIG S1 [file msphere.00676-21-sf001.pdf]

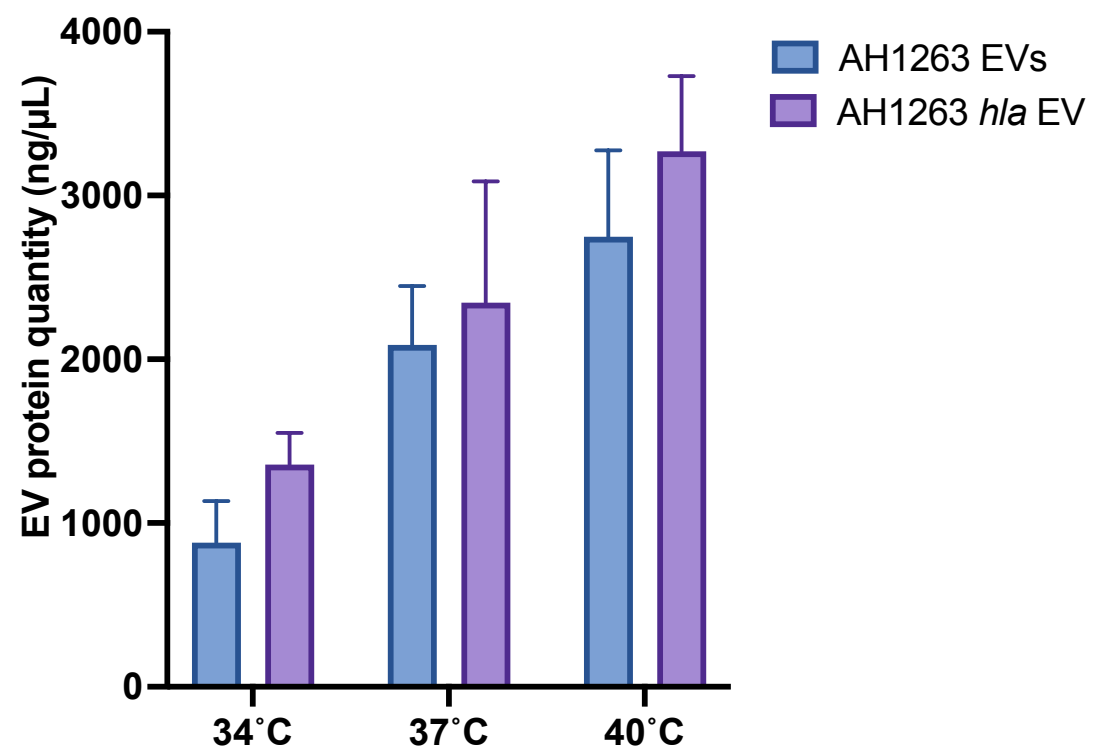

Supplement: FIG S2 [file msphere.00676-21-sf002.pdf]
